# Think Outside the Block: Rehabilitation Continuum After ACL Reconstruction with Adaptive Macro-Blocks—A Narrative Review

**DOI:** 10.3390/healthcare13192480

**Published:** 2025-09-29

**Authors:** Giandomenico Campardo, Roberto Ricupito, Carlotta Vercesi, Firas Mourad, Georgios Kakavas, Florian Forelli

**Affiliations:** 1 Campardo Private Clinic, via S. Marco 10, 33170 Pordenone, Italy; giandomenico.campardo@gmail.com; 2 Restart Physiotherapy, via Felice Grossi Gondi 35, 00162 Rome, Italy; robertoricupito90@gmail.com; 3 Centro Dello Sport—Swiss Olympic Clinica Ars Medica, 6925 Manno, Switzerland; carlotta.m.vercesi@gmail.com; 4 Department of Health, LUNEX University of Applied Sciences, 50, Avenue du Parc des Sports, 4671 Differdange, Luxembourg; 5 Luxembourg Health & Sport Sciences Research Institute A.s.b.l., 50, Avenue du Parc des Sports, 4671 Differdange, Luxembourg; 6 Facoltà Dipartimentale di Medicina e Chirurgia, Università Campus Bio-Medico di Roma, 00128 Rome, Italy; 7 Fysiotek Spine & Sports Lab, 17562 Athens, Greece; georgios.kakavas@gmail.com; 8 Haute-Ecole Arc Santé, HES-SO University of Applied Sciences and Arts Western Switzerland, 2800 Delemont, Switzerland; 9 Orthopaedic Surgery Department, Clinic of Domont, Ramsay Healthcare, OrthoLab, 95330 Domont, France; 10 Société Française des Masseurs—Kinésithérapeutes du Sport Lab, 93380 Pierrefite sur Seine, France

**Keywords:** anterior cruciate ligament, rehabilitation model, progression blocks, motor control, return to sport

## Abstract

**Background:** Conventional rehabilitation after anterior cruciate ligament reconstruction often follows a rigid, phase-based model. This structure may overlook individual differences in healing, neuromuscular control, and psychological readiness, leading to low return-to-sport rates and a high risk of reinjury. **Methods:** This narrative review proposes a flexible rehabilitation framework based on overlapping progression blocks. Inspired by principles of strength and conditioning, motor learning, and cognitive training, this model emphasizes continuous, individualized development instead of fixed timelines. **Results:** The proposed model integrates essential components—such as joint mobility, muscle activation, motor control, and psychological factors—throughout the entire recovery process. Functional testing is redefined as a dynamic and ongoing diagnostic tool that helps clinicians identify areas needing further development, rather than acting as a simple pass/fail gateway. Progression is guided by demonstrated readiness rather than time or phase completion. **Conclusions:** Rehabilitation using adaptive, overlapping progression blocks offers a more holistic and responsive approach. It allows for better personalization, supports safer decision-making, and improves the transition back to sport through sustained development of physical and cognitive capacities.

## 1. Background

Anterior cruciate ligament (ACL) injuries remain one of the most consequential and complex problems in sports medicine, particularly among young, physically active populations. The majority of ACL injuries occur in non-contact scenarios—most commonly during rapid deceleration, cutting maneuvers, or suboptimal landings in sport-specific contexts—with epidemiological reports reporting up to 68% non-contact incidence in adolescents and 55% in adults [1]. Surgical reconstruction followed by a structured rehabilitation program is widely accepted as the gold standard to restore knee function and mechanical stability [2]. Nevertheless, return-to-sport (RTS) outcomes remain below expectations [3]: approximately only 65% of individuals return to their pre-injury activity level, and fewer than 55% successfully resume competitive play [4]. These highlight a persistent discrepancy between intended clinical goals and real-world performance endpoints.

The determinants of this gap are multifactorial. Recovery of physical parameters alone is frequently insufficient, as psychological readiness exerts a critical influence on post-surgical performance and reintegration. More than 40% of patients report barriers such as fear of reinjury, loss of confidence, and kinesiophobia affecting successful RTS [5,6]. Longer-term studies further observed that a substantial proportion of individuals experience persistent symptoms or functional limitations, leading to lifestyle modification or reduction in sports participation [7,8,9,10]. Even in elite athletes—despite access to high-quality resources and multidisciplinary support—career longevity is often reduced, and performance tends to decline after injury, with reinjury rates remaining comparatively high [11,12]. Collectively, these data suggest that seemingly “clean” performance in controlled clinical settings does not automatically translate into functional robustness under the conditions of speed, fatigue, and unpredictability that characterize sport.

From a clinical and operational standpoint, these outcomes highlight the need for a framework that addresses both the biological healing and the recovery of functional capacities underpinning safe and effective RTS. Beyond graft biology and range of motion (ROM), athletes commonly require re-establishment of neuromuscular control and work capacity, followed by progressive acquisition of strength, power, and sport-specific skills. Critically, the recovery of these domains is rarely synchronous: trajectories are heterogeneous across individuals and over time, which challenges decision-making based solely on the time elapsed or formal “phase” completion. This heterogeneity supports the use of criterion-based, readiness-driven progression, where advancement reflects demonstrated ability rather than calendar milestones [13,14].

Arthrogenic muscle inhibition (AMI) is a central and often persistent constraint after ACL reconstruction (ACLR). AMI encompasses inhibitory processes following injury/surgery that depress voluntary quadriceps activation and alter motor strategies. Its effects are relevant not only in the early stages during open-chain exercises but also when the load increases and tasks progress to weight-bearing and single-limb drills [15,16,17]. Treating AMI as a longitudinal variable—monitored and managed throughout rehabilitation rather than “checked off” in the early period—provides a more realistic basis for aligning loading decisions, calibrating exposure to movement velocity, and selecting tasks as movement complexity escalates [15,16,17]. Particularly, accessible clinical signs and simple activation indices can help distinguish whether the limitations observed primarily reflect insufficient recruitment, strength deficits, or coordination problems, thereby improving the specificity of exercise prescription. Examples include a straight-leg raise without extension lag, superior patellar glide during quadriceps contraction, quadriceps surface EMG (sEMG) peak-activation patterns for the vasti and rectus femoris, and the central activation ratio (CAR) assessed via the superimposed burst technique [15,16,18,19,20]. These measures remain relevant beyond the initial post-operative period and should guide gradual exposure to more demanding tasks throughout the rehabilitation process [15,16,18,19,20].

One consequence of this perspective is the redefinition of functional tests. Rather than serving as a rigid pass/fail pass between predefined stages, testing is most valuable when used as diagnostic feedback—a series of repeated, informative windows that guide clinical decision-making. In this view, suboptimal results are interpreted as targets for development, directing focused work on activation, neuromuscular control, strength, or coordination in parallel with the acquisition of new abilities instead of functioning as obstacles that halt broader progression [15,19,20,21,22,23]. Therefore, readiness resulting from repeated assessments can guide dosage, task selection, and rate of progression in order to respond to the athlete’s evolving profile [15,18,19,20,21,22,23,24,25,26].

## 2. Methods

This narrative review was conducted to explore and synthesize current evidence, theoretical models, and clinical practices related to ACLR rehabilitation, with a focus on developing an adaptive, macro-block-based continuum. The methodology followed a structured but non-systematic approach, allowing for both scientific evidence and expertise to inform the conceptual framework.

### 2.1. Literature Search Strategy

A targeted literature search was performed between January 2025 and March 2025 using PubMed, Scopus, and Web of Science databases. Search terms included combinations of the following:

- “ACL reconstruction”, “ACLR rehabilitation”, “return to sport”,
- “arthrogenic muscle inhibition”, “motor learning”, “neurocognitive training”,
- “non-linear periodization”, “functional testing”, “criterion-based progression”.

Boolean operators (AND/OR) were applied to refine search queries, and reference lists of key articles were manually screened for additional relevant sources. Only articles published in English or French were included. Priority was given to systematic reviews, clinical practice guidelines, high-quality cohort studies, expert clinical commentaries, and consensus statements. Studies that specifically addressed neuromuscular deficits, motor control, strength and conditioning principles in rehabilitation, and RTS criteria after ACLR were selected.

### 2.2. Selection and Synthesis

Relevant publications were screened based on title and abstract by the lead author. Full texts were reviewed when the content addressed one or more of the following themes:

- Limitations of linear, phase-based ACLR rehabilitation
- The role of AMI and neuromuscular inhibition
- Periodization strategies adapted to rehabilitation
- Testing as a dynamic diagnostic tool
- Functional and cognitive demands in RTS preparation

Articles were then categorized into thematic clusters corresponding to the conceptual building blocks of the proposed continuum. These clusters were cross-referenced with field experience and expert knowledge from physiotherapy, orthopedics, and sports science to refine the model.

### 2.3. Clinical Integration

The review includes empirical insights from clinical practice in various rehabilitation settings: private practices, sports medicine centers, and academic environments. The concepts have been tested in real-world scenarios, including progressive exercise prescription, functional testing, and return-to-play (RTS) decision-making processes. Multidisciplinary discussions among the co-authors guided the refinement of the model, ensuring its clinical feasibility and adaptability to different patient profiles.

This methodology enabled the development of a clinically grounded, evidence-informed rehabilitation continuum that bridges the gap between theory and practice while challenging traditional paradigms.

### 2.4. Limitations

As this work is a narrative review rather than a systematic one, the selection of studies may be subject to bias. This limitation should be considered when interpreting the findings.

## 3. Critique of the “Traditional” Phase-Based Model

The conventional ACLR rehabilitation protocol is typically divided into four or five distinct phases (e.g., early, mid, late, on-field), each with specific clinical objectives and therapeutic goals [21,27,28]. Progression between these phases is generally determined by the successful completion of predefined clinical and functional criteria, assessed via standardized testing with predetermined cut-off values [21,24,27,28,29,30].

Periodization refers to the systematic planning and modulation of training variables such as load, volume, and repetition schemes, goals to enhance physiological adaptations while managing fatigue and tissue overload [13,14,31,32,33]. In traditional ACLR rehabilitation (Table 1), periodization often aligns with the linear phase structure, with a gradual and progressive increase in load and intensity across defined stages [14]. While progressive overload is fundamental for strength development, the systematic variation characteristic of periodized programming is demonstrably more effective than non-periodized approaches for improving physical qualities like strength, endurance, and power [14,32]. Alternatively, non-linear (or undulating) periodization provides greater adaptability by incorporating frequent variations within and across phases, thereby allowing clinicians to tailor progression to the individual patient’s response and readiness [13,31].

While the linear, phase-based structure provides a clear roadmap, it presents notable theoretical and practical limitations [13,31]. Its sequential organization can lead to fragmented motor skill development, where completing one phase may prematurely discontinue exercises from previous stages, thereby disrupting training continuity, delaying recovery, and compromising preparedness for more demanding tasks [32,33]. Secondly, the strict focus on isolated capacities within each phase can permit progression even when the athlete has not yet developed the robust foundational abilities required to safely and effectively manage the demands of the subsequent stage [14,32,34]. Furthermore, the traditional model struggles to clearly integrate key performance concepts such as the strength continuum (e.g., differentiating eccentric explosive strength for deceleration from isometric strength for tendon stiffness) and lacks standardized frameworks for the incorporation of neurocognitive training [13,14,31,34].

## 4. Proposal: The Rehabilitation Continuum of Overlapping Macro-Blocks

We propose replacing rigid, sequential phases with a dynamic rehabilitation continuum organized in overlapping macro-blocks. This model synthesizes principles from strength and conditioning (periodization), motor learning, neurocognitive training, the control-to-chaos continuum, and the gym-to-field transition [13,14,31,32,33,34,35,36,37].

Macro-blocks are broader periods that preserve and re-dose foundational elements (e.g., management of AMI and motor control) while progressively layering strength, power, and sport-specific skills [38,39]. Progression is readiness-driven, not time-driven, allowing clinicians to tailor pace and emphasis to biological and psychological responses and functional needs, while overlap prevents stop–start losses and supports coherent skill development—consistent with non-linear periodization [27,40,41,42,43].

In parallel, we introduce preparatory work early to build capacities expected to underpin upcoming demands—even before they are formally assessed—thereby conditioning the patient structurally and neuromuscularly for more complex tasks and higher loads. This proactive layering increases confidence in readiness and facilitates safer, smoother progress toward advanced function and sport-specific performance [13,14,36,37,42,44,45,46,47].

## 5. Testing to Unlock New Abilities, Not Just a Passport for a New Phase

In the proposed rehabilitation framework, the role of functional and performance evaluation is fundamentally redefined relative to conventional approaches. Testing is no longer conceptualized as a singular, static “one-off session” primarily intended to sanction progression between rigidly predefined phases [21,24,25,27,28]. Instead, it is reframed as a continuous, hypothesis-driven assessment process distributed across flexible temporal windows [14,26,48,49,50]. Within these windows, the underlying clinical assumption is that the patient has achieved a minimum competence threshold sufficient to allow the safe introduction of novel motor and functional capacities [24,26,49,50].

Accordingly, the execution of specific tests within these intervals should not serve as a simple inclusion/exclusion gate for phase-based advancement. Rather, test outcomes—particularly suboptimal results—provide the clinician with essential diagnostic information. These qualitative and quantitative data directly guide the identification of specific neuromuscular, strength, or motor-control capacities that require further targeted development within the individualized rehabilitation program [21,24,25,26].

Assessment within this continuum is best understood as an ongoing, adaptive process occurring within flexible timeframes. During these intervals, competence thresholds permit the acquisition of new abilities while deficits are addressed in parallel; test results are therefore not rigid gatekeeping mechanisms but diagnostic signals that highlight where to focus remediation without halting development in other domains. This organization supports continuous advancement toward functional goals and reduces stop–start patterns that can arise when progression depends solely on phase completion [21,24,25,26,49,50]. Conceptually, this reframing answers two practical questions for clinicians: (1) Which critical aspects of rehabilitation—e.g., persistent neuromuscular deficits—may be insufficiently addressed by current protocols? (2) Where is the patient currently positioned within the process, and which abilities require immediate development to optimize subsequent steps? In this sense, tests are best viewed as “windows of opportunity”, not merely a “passport” to the next stage.

## 6. The Testing Continuum: From Foundational Control to Sport-Specific Chaos

### 6.1. Foundational Phase (Joint Health and Neuromuscular Reactivation)

Before effective ground interaction can occur, it is essential to first establish an optimal local environment by controlling swelling and pain—critical for minimizing spinal reflex inhibition—and to restore basic neuromuscular function, including full passive extension and sufficient flexion (e.g., 130°), as these are prerequisites for efficient, resistance-free muscle contraction [15,21,22]. Accordingly, early recognition and treatment of AMI is critical [18]. AMI is a protective reflex that inhibit muscle activation after injury or surgery [17], arising from altered afferent input associated with ACL injury and reconstruction [15,22,23]. This disruption leads to sensorimotor uncertainty and imprecise sensory predictions, clinically manifesting as inflammation, swelling, altered afferent signals (including sensory overloading and deafferentation), motoneuron inhibition, increased antagonist co-contraction, altered spinal reflexes excitability, and ultimately, impaired voluntarily quadriceps recruiting [44,51,52].

**Figure 1:**
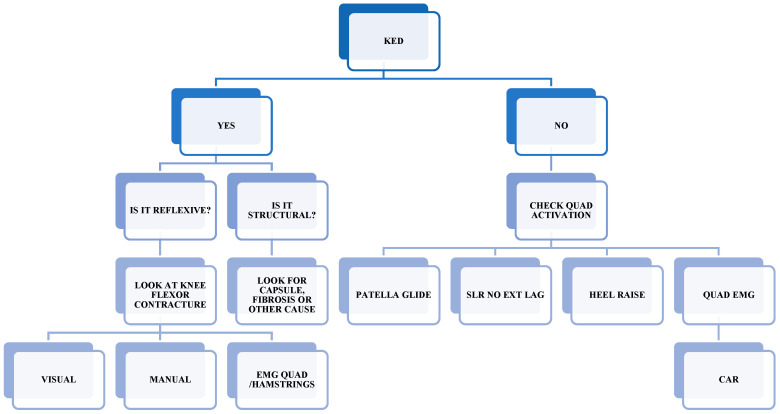
Algorithm for the assessment and treatment of AMI. Common clinical indications for quadriceps recovery are patella superior glide during quadriceps contraction, straight leg raise with no extension lag, heel raise from floor during quadriceps contraction, symmetry in quadriceps sEMG peak activation for vasti and rectus femoris, and central activation ratio [15,16,18,22]. Note: KED: knee extension deficit, sEMG: electomyography, SLR: straight leg raise, EXT: extension, QUAD: quadriceps, HAM: hamstrings, CAR: central activation ratio.

In the early assessment period (first 6 weeks), differentiating a true structural knee extension deficit from a reflex flexor contracture or other structural limitations is crucial [18]. We propose the use of sEMG to quantify hamstring–quadriceps co-contraction activity when a flexor reflex is suspected [22]. If no contracture is present, assessing peak and/or Root Mean Square (RMS) sEMG (quantification of muscle activation) activity of the quadriceps components can help detect compensatory shifts towards predominant rectus femoris and/or vastus lateralis activation [15,18,19,22]. From week 6 onwards, additional measures are recommended, including central activation ratio (CAR) via the superimposed burst technique, isometric strength tests at 60° and 90° of knee flexion, and continued sEMG monitoring [19,20]. Clinical indicators of recovery include palpable superior glide of the patella during contraction, absence of extension lag during straight leg raise, heel raise during quadriceps contraction, symmetrical quadriceps sEMG peak activation (across the vasti and rectus femoris), and normalization of the CAR [15,16,18,20] (Figure 1).

Central activation ratio (CAR) is obtained with the superimposed burst technique during an isometric knee-extension MVC (hip ~90°, knee 60–90°): at the torque plateau a brief supramaximal tetanic stimulus is delivered to the femoral nerve/quadriceps motor points; any evoked torque increment indicates incomplete voluntary activation (central activation failure/AMI). Clinically, CAR is used to detect quadriceps under-activation after ACLR, track recovery of neural drive, and inform load and plyometric/running progression alongside pain, ROM, and strength metrics.

**Figure 2:**
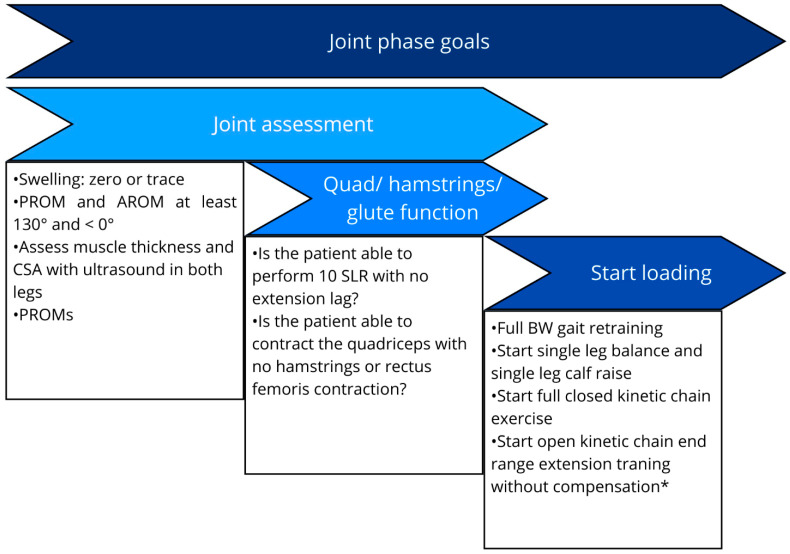
Key performance indicators for joint phase assessment before starting weight bearing exercise [21,29,30,53,54,55]. Note: AROM; avtive range of motion, PROM; passive range of motion, SLR; straight leg raise; BW body weight; * start open kinetic chain based on previous recommendation [30,53,56].

Beyond ROM and quadriceps activation, early stimulation of musculature supporting the pelvis, hip, leg, and foot is essential to prevent deconditioning and neuromuscular control deficits. Assessment in this phase should therefore consider not only targeted quadriceps retraining but also the patient’s capacity to manage proximal and distal joints effectively. Figure 2 summarizes the key performance indicators for this phase.

### 6.2. Neuromuscular Control and Work Capacity Phase

The prerequisite before advancing significantly into strength training is restoring robust motor control during closed kinetic chain (CKC) exercises and development of sufficient work capacity [57,58,59]. This is vital for building both muscular endurance and maximal isotonic/isometric strength prior to introducing more dynamic and reactive movements [57,58,60]. Alterations in weight distribution and movement kinematics during fundamental tasks such as squats and lunges can transfer into dynamic activities, thereby potentially increasing the long-term risk of osteoarthritis or reinjury [60,61,62].

**Figure 3:**
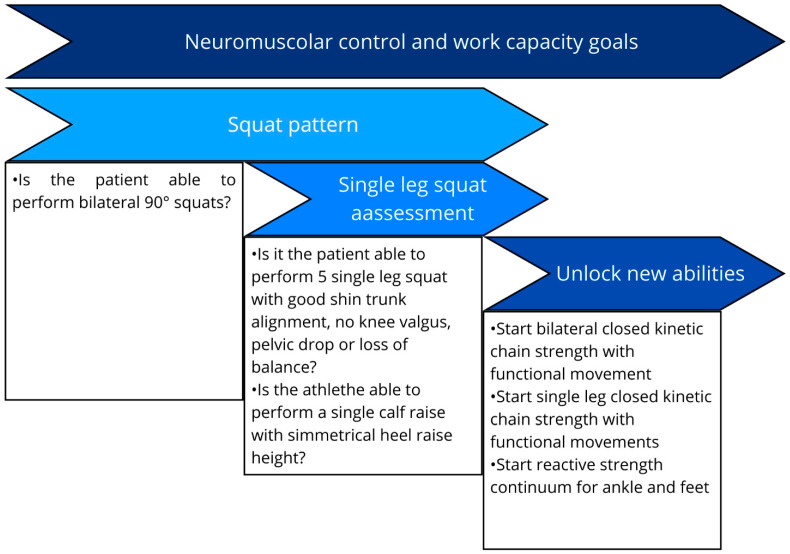
Neuromuscular control phase key performance indicators before starting strength training [17,28,63,64,65,66,67]. Note: BW body weight, PROM: passive range of motion, AROM: active range of motion.

Assessment during this phase should include evaluating neuromuscular control through functional tests such as single-leg squat quality assessment and the Y-balance test, as well as work capacity using step-down tests, before commencing intensive strength training [17,28,63,64]. Assessing heel raise height is also important to determine whether the foot/ankle complex possesses the capacity for full plantarflexion—a prerequisite for initiating endurance-oriented resistance training of the calf musculature [65,66,67]. Before initiating a structured strength training program, and in addition to ensuring appropriate load progression and exposure to diverse movement patterns, we recommend achieving specific performance indicators. This enhances clinicians’ awareness of neuromuscular baseline capacity and ensures safer progression when advancing load intensities (Figure 3).

### 6.3. Strength Development Phase

The primary goal before progressing to running and fast tensile load activities is establishing sufficient endurance and maximal strength. With motor control already consolidated in the previous phase, this macro-block emphasizes the progressive loading of the limb to develop the foundation required for dynamic movements [68,69,70].

Assessment of strength is essential. A detailed description of specific tests is provided in Table 2. Continuous monitoring of joint health and neuromuscular control from earlier phases must be maintained, and it is equally crucial to ensure that progressive reactive foot-strength training has been initiated [28,71,72].

### 6.4. Power and Linear Running Phase

Just as work capacity prepares muscle for subsequent strength development, a progressive running program is essential for connective tissue adaptation. A well-structured running program conditions the knee joint to tolerate rapid loading and high vertical ground reaction forces (vGRF) before advancing to more demanding tasks such as deceleration and pivoting, which require substantial muscle–tendon capacity to effectively manage these stresses. The prerequisite for introducing change of direction (COD) is the achievement of the strength goals established in the previous phase [27,82,83]. This progression unlocks the transition to explosive exercises—including eccentric, isometric, and concentric rate-of-force development, as well as linear and curvilinear running. The aim of this macro-block is to restore the capacity to withstand high tensile loads and to perform energy storage and release activities that are fundamental for acceleration and deceleration. Importantly, this is the phase where gym-based and field-based training begins to be systematically integrated [24,84,85].

Velocity-based training is a relevant method in ACLR rehabilitation to restore explosive strength and running-specific capacities. By emphasizing movement velocity rather than maximal load, velocity-based training facilitates the development of muscular power and neuromuscular coordination—qualities that are critical for acceleration, change of direction, and sprinting. When introduced during the intermediate or advanced phases of rehabilitation, it allows for a gradual reintroduction of explosive running tasks while minimizing mechanical stress on the reconstructed joint [86,87,88].

Assessment in this phase focuses on the athlete’s ability to absorb and produce rapid vertical and horizontal forces. This is typically evaluated through jump tests such as double-leg countermovement jump (DLCMJ), single-leg countermovement jump (SLCMJ), double-leg drop jump (DLDJ), and single-leg drop jump (SLDJ). Parameters of interest include jump height, Limb Symmetry Index (LSI), Reactive Strength Index (RSI), and ground contact time (GCT) [29,89,90,91]. We recommend not relying solely on outcome metrics (e.g., jump height) but also considering kinetic and kinematic strategies, such as second impulse (the late propulsive vertical impulse, which indexes distal/ankle plantarflexor contribution and push-off sequencing) and force at zero velocity (the vertical ground reaction force at the instant the COM’s vertical velocity equals 0, which indexes eccentric–concentric coupling stiffness and readiness to re-accelerate).

**Figure 4:**
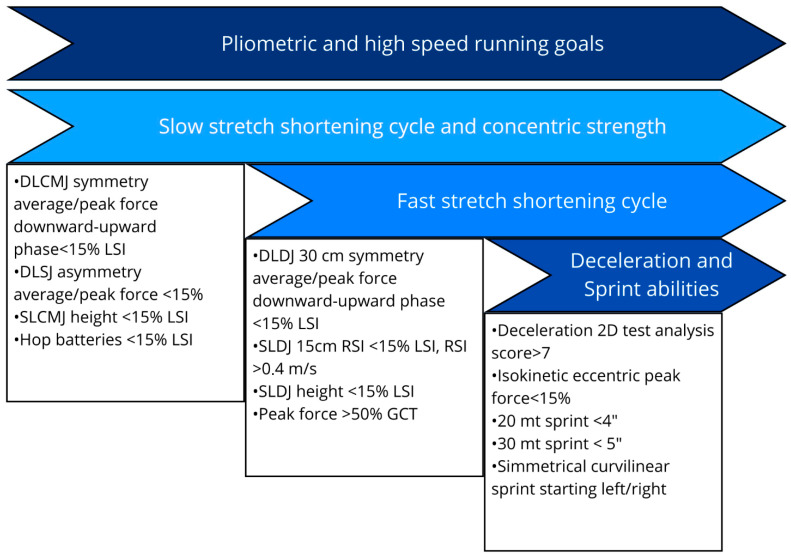
Key performance indicator before starting planned change of direction and multidirectional speed training [92,93]. DLCMJ: double-leg countermovement jump, SLCMJ: single-leg countermovement jump, DLDJ: double-leg drop jump, SLDJ: single-leg drop jump, 2D: two dimensions, LSI: Limb Symmetry Index (injured/healthy × 100), GCT: ground contact time, RSI: Reactive Strength Index, mRSI: modified reactive strength index.

Achieving these targets, in combination with progressive loading, allows for the safer reintroduction of complex motor tasks such as change of direction and repeated sprint efforts, by enhancing neuromuscular control and reducing overload risk [84]. As previously highlighted, exposure and progression through different strength modalities—maximal, explosive, and reactive—applied across multiple planes and vectors are essential for developing advanced motor skills such as change of direction. Concurrently, meeting predefined performance indicators (Figure 4) enables clinicians to determine whether the patient has achieved the minimum neuromuscular thresholds necessary to tolerate the demands involved in change of direction tasks.

A common rehabilitation goal is achieving an LSI greater than 85% across relevant strength tests, in addition to assessing symmetry in SLCMJ downward-phase impulse, velocity, and force as well as isokinetic strength. The downward phase comprises unweighting followed by eccentric braking, terminating at the instant of minimum COM height; it establishes the mechanical initial conditions of the subsequent concentric push-off. Clinicians should monitor for compensatory braking strategies—such as excessive trunk lean and reduced knee flexion during deceleration tests—linking these observations to a deficit in eccentric strength capacity and to the force–time characteristics of the CMJ [94,95,96].

### 6.5. Change of Direction Phase (Planned)

The prerequisite before transitioning to unplanned, fast, multidirectional movement is development of the capacity to effectively brake, absorb, and produce force with minimal ground contact time and maximal effort—a particularly important consideration given that eccentric quadriceps forces can reach up to six times body weight during cutting maneuvers [95,97,98,99].

**Figure 5:**
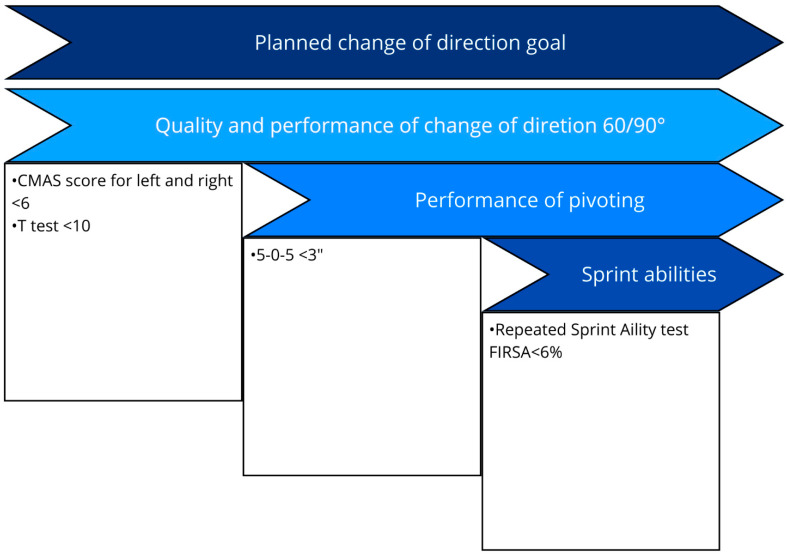
Key performance indicator before starting agility training [100,101,105,106,107]. CMAS; Cutting Movement Assessment Score; FIRSA: fatigue index repeated sprint ability.

Performance should be assessed in planned COD tests such as the 505 test and the pro-agility test, supplemented by qualitative movement analysis using instruments like the Cutting Movement Assessment Score (CMAS). In addition, assessing fatigue resistance during repeated-sprint ability tests is crucial, while continuous monitoring of assessments introduced in earlier phases should be maintained [100,101]. We propose that kinetic assessment during tests such as the CMAS provides clinically meaningful insight into knee loading patterns. This approach offers a more functional perspective than simply dichotomizing biomechanics into “high” versus “low” risk and also helps determine whether the patient is underloading the knee due to fear or diminished functional capacity [100,102,103,104]. Before initiating unplanned change of direction or agility tasks, it is essential to ensure that the patient has been sufficiently exposed to all COD variations at different velocities and has achieved the key performance indicators (Figure 5).

Progression within this phase begins with planned COD drills, including maneuvers like 60° crossover cuts, 90° sidesteps, and 135–180° pivot cuts.

### 6.6. Agility and Chaos Phase (Unplanned)

The ultimate prerequisite is readiness to tolerate the combined cognitive demands and tissue loading required in competitive sport [16,84]. Transitioning to reactive, unpredictable movements requires not only robust physical capacity but also efficient perceptual–cognitive processing [37,42,43].

Assessment in this final macro-block focuses on performance under unpredictable stimulus conditions. This includes reactive COD tests and sport-specific drills that simulate competitive scenarios (e.g., “11 to Perf Score”) [101]. As in earlier phases, a gradual increase in exposure, frequency, speed, and stimulus intensity is essential for assessing the patient’s ability to tolerate reactive and unpredictable movement challenges. Achieving predefined performance targets prior to return to training provides both clinician and patient with greater confidence in neuromuscular and physical readiness. The KPIs to be achieved during an unplanned COD test are the same as those to be achieved during a planned COD; see Figure 5.

Progression involves introducing increasingly chaotic scenarios—such as small-sided games or drills with multiple unpredictable stimuli—but only after the athlete has demonstrated proficiency in both planned COD and reactive testing. Utilizing GPS tracking can help confirm that the athlete achieves acceleration, sprint, and deceleration values that are comparable to match-play or training demands during on-field rehabilitation [108].

**Figure 6:**
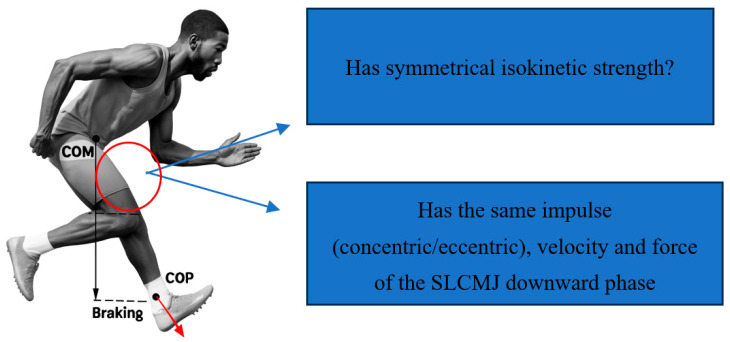
Compensation strategy from knee to hip/trunk. COP: center of pressure; COM: center of mass; con; concentric, exc; excentrique, SLCMJ; single-leg countermovement jump.

This testing continuum ensures that athletes acquire the prerequisite abilities before progressing to more demanding tasks, thereby moving beyond simple time-based or phase-completion criteria. Each rehabilitation session should be considered an opportunity to observe movement quality and monitor progress towards goal achievement, thereby guiding the ongoing, individualized process. Accordingly, we emphasize the use of monitoring technologies (e.g., sEMG, force plates) during exercises to ensure that tasks function not merely as repetitive drills but as learning opportunity to develop correct motor patterns, while preventing both inter-limb and intra-limb compensation strategies. For example, if an unclear braking strategy is observed during a deceleration test (e.g., excessive forward trunk lean during the penultimate step), it becomes necessary to assess eccentric strength capacity using isokinetic testing and to evaluate the downward force–time characteristics of the CMJ [108] (Figure 6). Such analyses help determine whether the patient possesses adequate neuromuscular control and strength qualities to sustain rapid horizontal decelerations. Furthermore, given the inherent variability of sport movement, movement quality tests should be interpreted with the aim of discriminating whether the patient is effectively utilizing the knee to absorb load or instead relying on compensatory strategies [87].

Table 3 provides a one-page summary of the rehabilitation continuum by overlapping macro-blocks. For each phase it lists the following: prerequisites, core tests/metrics used to monitor progression, and explicit go/no-go clinical gates guiding advancement. Acronyms are defined below the table.

**Figure 7:**
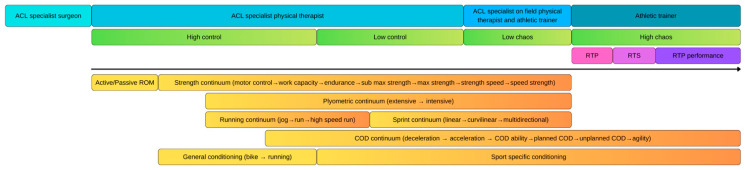
New periodization model of ACL Rehabilitation.

## 7. Visual Representations and Clinical Implications

In clinical practice, this framework positions readiness as the primary driver of progression while preserving previously acquired gains. Testing is organized into regular time windows (≥4 weeks) and interpreted as iterative diagnostic feedback, yielding a three-level classification—passed/partially passed/not passed—that guides decisions regarding what to introduce, what to maintain, and when to hold.

Because macro-blocks overlap, advancing does not entail abandoning prior work. Foundational elements are retained, re-dosed, and integrated as higher-level abilities are layered, while targeted interventions for residual deficits are incorporated within the same session—typically in the preparatory segment and, when appropriate, in a brief bridging segment—without reliance on fixed time prescriptions.

For example, during a phase dominated by high-intensity plyometric work (e.g., CMJ or tuck jumps), it is essential to include preparatory activation drills within the warm-up that recall elements from the joint-control phase. These may include short-duration isometrics or co-contraction tasks monitored with sEMG, aimed at priming motor cortex activity and reinforcing selective muscle group engagement, while limiting compensatory activation patterns. Additionally, incorporating motor control exercises with external feedback or motor imagery techniques serves both as a preparatory strategy and as a bridge to advanced skills such as change of direction mechanics [109]. Cognitive activation drills may also be included to enhance neuromotor responsiveness [110].

The main body of the session may then include intensive plyometric drills, one or two exercises targeting strength or strength–speed qualities, a muscular endurance component, and targeted kinetic chain exercises (e.g., calf, hip abductors, and gluteal musculature). Finally, the cooldown phase can integrate gamified neuromotor activities or virtual reality tools to support both engagement and cognitive–motor integration. By continuously cycling earlier-phase elements into higher-level sessions, this model ensures that patients preserve achieved neuromuscular, cognitive, or technical gains. Rather than regressing, patients are able to navigate the entire spectrum of recovery as an integrated, functional continuum (Figure 7).

By way of illustration, consider a post-ACLR patient who reaches the end of the joint phase with satisfactory results on the exit criteria, except for active knee extension, which remains below that of the contralateral limb. At the first testing window, this profile is classified as partially passed: exposures from the next macro-block are introduced, while the warm-up or preparatory segment of each session prioritizes targeted work to progressively restore active knee extension. This focus is maintained until the specific criterion is achieved. The main part of the session develops the competencies of the current block, and brief bridging elements at the end may be used to consolidate quality. At the subsequent window, readiness is re-evaluated against the same criteria; once active extension meets the standard, the window is considered passed, progression within the new block is expanded, and a small, purposeful exposure to the previously targeted element is retained to avoid stop–start losses.

In another example, several months after surgery a post-ACLR patient demonstrated a quadriceps maximal strength deficit and incomplete passive knee flexion. At the first testing window this configuration is likewise classified as partially passed: weekly sessions are structured so that the preparatory segment addresses these unresolved items from the previous block, while the main segment develops the abilities specific to the current block. The targeted work for residuals is distributed within the same session according to intensity demands, without reliance on present duration for each component. At the next window, the same criteria are re-applied; once the strength and range benchmarks specified in the manuscript are achieved, the window is passed, progression within the current block is broadened, and essential elements from the prior block are deliberately re-dosed to preserve adaptations already acquired.

Across the continuum, elements often under-addressed by rigid-phase models are integrated continuously: arthrogenic muscle inhibition is monitored longitudinally; progressive neurocognitive loading reflects perceptual–decision demands; and the control-to-chaos framework structures the re-introduction of sport-relevant tasks. The model places equal emphasis on numeric thresholds and the quality of movement used to reach them. Quantitative signals are interpreted together with qualitative analysis of execution—considering neuromuscular strategies and compensatory patterns—to ensure that progression is both meaningful and safe, with task-rich appraisal preferred over single-task inferences where appropriate.

Operationally, this cadence benefits from a shared, ability-centered language. Surgeons, physical therapists, and coaches coordinate around achieved milestones and capabilities, clarifying why a new exposure is introduced, which prior abilities are deliberately maintained, and which residuals are addressed in parallel—making day-to-day practice more coherent, efficient, and directly tied to on-field demands.

## 8. Conclusions

The traditional phase-based approach to ACLR rehabilitation, while offering structure, may inadvertently impede optimal recovery by fostering compartmentalized skill development and permitting premature progression without sufficient foundational capacity. We advocate for a paradigm shift towards a rehabilitation continuum, defined by overlapping macro-blocks and guided by criterion-based testing that targets the acquisition of specific requisite abilities. For example, during the power phase, we should not abandon testing and exercise addressing AMI. In this context, the warm-up can be used to revisit key elements from earlier phases. The central part of the session should emphasize current priorities (e.g., plyometric training), while the final portion can reinforce competencies from previous stages (e.g., endurance strength). This approach ensures that the patient reserves and consolidates skills already acquired. This model promotes continuous development, individualization, and the integration of principles from motor learning, neurocognitive science, and strength and conditioning. Within this framework, testing functions not as a rigid gatekeeper between phases but, together with structured exercise progression, as a dynamic diagnostic tool for assessing readiness to meet increasingly complex functional demands—ranging from managing AMI and restoring basic control to executing reactive, sport-specific movements under chaotic, game-like conditions.

## Figures and Tables

**Table 1:** Actual periodization after ACLR.

Macrocycle							
1 Mesocycle (Early After ACLR)		2 Mesocycle (Mid Phase After ACLR)		3 Mesocycle (Late Phase After ACLR)		4 Mesocycle (on Field Phase After ACLR)	
Weeks 1–3	Weeks 3–6	Weeks 6–9	Weeks 9–12	Weeks 12–15	Weeks 15–18	Weeks 19–21	Weeks > 22
Focus on ROM and quadriceps reactivation	Focus on gait and work capacity	Focus on hypertrophy and strength	Focus on strength and power	Focus on running and SSC	Focus on COD	Focus con unplan COD	Focus on agility

Note: ACLR; anterior cruciate ligament reconstruction, ROM; range of motion, SSC; stretch-shortening cycle, COD; change of direction.

**Table 2:** Key performance indicator goals before starting power and running training [50,64,73,74,75,76,77,78,79,80,81].

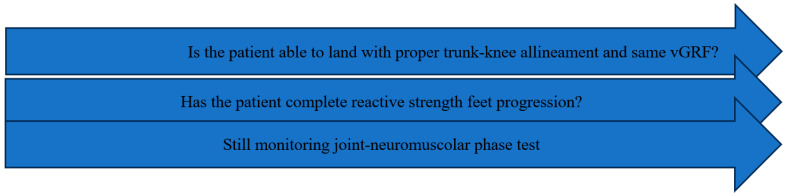		
Movement	Goals	Movement Prerogative
Single-leg press 90° with hip flexed at 45°	1.25 BW for 8 rep	Enough muscle–connective tissue isotonic strength to absorb and release vGRF
Single-leg bridge endurance test	>20 rep or less than 5 rep difference b/t leg with good quality	Enough hip endurance and lumbopelvic capacity to control pelvic stability and hip propulsion
Single-leg calf raise endurance test	>20 rep or less than 5 rep difference b/t leg with good quality and heel raise height	Enough calf endurance to control propulsion during late stance
6 RM calf raise	0.5 BW 6 rep with good quality	Enough calf isotonic strength to absorb and release vGRF
6 RM seated calf raise	1.5 BW 6 rep with good quality	Enough calf isotonic strength to absorb and release vGRF and control anterior tibial translation
Isometric knee extension 60°	>70% LSI, >2.00 Nm/kg	Enough muscle–connective tissue isometric strength to control the amortization phase of ground contact time
Isometric knee extension 90°	RTD > 70% LSI	Enough muscle–connective tissue isometric explosive strength to control the amomortization phase of ground contact time
Isokinetic 60° knee extension	1.45 Nm/kg or >70% LSI	Enough muscle–connective tissue isokinetic strength to control knee flexion movement
Isometric knee flexion 60°	>70 LSI	Enough muscle–connective tissue isometric strength in order to control tibial translation
Single-leg squat raise	>85% LSI, >10 rep	Enough quadriceps endurance to control deceleration of the COM during stance phase
Isometric single-leg squat	Peak force > 1.5 BW	Enough isometric strength to control the amortization and prepare leg stiffness during the phase of ground contact time
Normal ankle motion	>25° passive dorsiflexion >65° FLA dorsal extension	Enough ankle ROM and first toe for terminal stance—propulsion
Hip abductor to BW ratio	>33%	Enough abductor muscle strength to control pelvic movement in frontal plane

Note: Rep: repetition, b/t: between, BW: bodyweight, Nm: Newton × meters, kg: kilograms, RTD: rate of torque development, vGRF: vertical ground reaction force, COM: center of mass, FLA: flexor longus allucis, LSI: Limb Symmetry Index, ROM: range of motion.

**Table 3:** Rehabilitation continuum by overlapping macro-blocks: prerequisites, core assessments, and go/no-go clinical gates.

Phase	Prerequisites	Core Tests/Metrics	Go/No-Go Clinical Gate
(a) Foundational—Joint Health And Neuromuscular Reactivation	Pain/swelling controlled; full passive extension; adequate flexion (~130°); early AMI management to limit reflex inhibition [15,17,18,21,22,23].	sEMG hamstrings–vastus co-contraction (if reflex suspected); quadriceps peak/RMS sEMG (VL/VM/RF); from week 6 add CAR (superimposed burst) and isometric strength at 60°/90°; continue sEMG [15,18,19,20,22].	Superior patellar glide; no SLR lag; heel raise with quadriceps contraction; symmetrical quadriceps peak sEMG; CAR normalized [15,16,18,20] (Figure 1 and Figure 3).
(b) Neuromuscular Control And Work Capacity	Robust CKC motor control and sufficient work capacity; correct aberrant load distribution/kinematics before high-intensity strength [57,58,59,60,61,62].	Single-leg squat quality; Y-Balance; step-down capacity; heel raise height (full plantarflexion readiness for calf endurance loading) [17,28,63,64,65,66,67].	Advance strength when CKC control/work capacity (and heel raise) criteria are met and load progression is safe (Figure 4) [17,28,63,64,65,66,67].
(c) Strength Development	Endurance and maximal strength established; maintain joint health/neuromuscular work; initiate/continue reactive and foot-strength training [28,68,69,70,71,72].	Strength assessment per Table 2 (standardized isometric/isokinetic measures at 60°/90°).	Begin running only after Table 2 strength targets are achieved. (Table 2)
(d) Power And Linear Running	Progressive running for connective tissue conditioning and high vGRF tolerance; introduce COD only after strength goals; integrate gym + field; consider VBT for power at lower joint stress [24,27,82,83,84,85,86,87,88].	DLCMJ, SLCMJ, DLDJ, SLDJ; metrics: jump height, LSI, RSI, GCT; add kinetic/kinematic strategies (second impulse, force at zero velocity) [29,89,90,91].	Move to planned COD when phase targets are met (Figure 5) with symmetry in SLCMJ downward-phase impulse/velocity/force and adequate eccentric/isokinetic strength [94,95,96]. (Figure 5)
(e) Change of Direction (Planned)	Effective braking/force absorption with minimal GCT at high effort; eccentric quadriceps forces during cutting up to ~6 × BW [95,97,98,99].	Planned COD tests (505, pro-agility) + CMAS; repeated-sprint ability; kinetic-informed interpretation to detect knee under-loading from fear/reduced capacity [100,101,102,103,104].	Proceed to unplanned COD/agility after exposure to all variants across velocities and KPI attainment (Figure 6) [100,101]. (Figure 6)
(f) Agility And Chaos (Unplanned)	Readiness for combined perceptual–cognitive demands and tissue loading of competitive sport [16,37,42,43,84].	Reactive COD and sport-specific drills (e.g., “11 to Perf Score”); corroborate on-field external load profiles with GPS (acceleration, sprint, deceleration) [101,108].	Advance toward return to training when predefined targets are achieved and movement quality shows effective knee load absorption (no compensatory under-loading) [87]. (Figure 7)

AMI = arthrogenic muscle inhibition; sEMG = surface electromyography; CAR = central activation ratio; CKC = closed-kinetic chain; LSI = Limb Symmetry Index; RSI = Reactive Strength Index; GCT = ground contact time; VBT = velocity-based training; COD = change of direction; CMAS = Cutting Movement Assessment Score; vGRF = vertical ground reaction force; DLCMJ/SLCMJ/DLDJ/SLDJ = double/single-leg countermovement/drop jump; SLR = straight-leg raise; RF = rectus femoris; VL/VM = vastus lateralis/medialis.

## Data Availability

No new data were created or analyzed in this study.
